# Comparison of injury pattern and clinical outcomes between young adults and elderly patients with alcohol-related injury in South Korea 2011–2016

**DOI:** 10.7717/peerj.7704

**Published:** 2019-09-27

**Authors:** Jae Hee Lee, Duk Hee Lee

**Affiliations:** Department of Emergency Medicine, Ewha Womans University, Seoul, South Korea

**Keywords:** Elderly, Alcohol, Injury, Mortality, EMR-ISS

## Abstract

**Background:**

Alcohol is an important factor that contributes to emergency department (ED) visits due to injury. However, the role of alcohol in elderly patients visiting ED due to injury has not been clearly defined. This study aims to examine age and alcohol as risk factors of injury severity and clinical outcomes.

**Methods:**

This study included patients who visited EDs between January 2011 and December 2016. Data was obtained from the Emergency Department-Based Injury In-depth Surveillance of the Korea Centers for Disease Control and Prevention, South Korea. Injury patients aged ≥18 years were included, but those who visited the ED more than 48 hours after injury, with unknown clinical outcomes (admission, mortality, and excess mortality ratio-adjusted injury severity score [EMR-ISS]) were excluded.

**Results:**

We analyzed 887,712 patients, of whom 131,708 (17.7%) non-elderly and 9,906 (7.0%) elderly had alcohol-related injury. Falls and slips are the most common injury mechanism (37.9%) in patients consuming alcohol (36.3% non-elderly/58.40% elderly). The injury occurred on roads (40.6%), houses (33.8%), and commercial facilities (11.9%) in elderly patients consuming alcohol. Suicide rate was 12.0% in elderly and 9.7% in non-elderly patients. According to the time of day of injury, evening (60.8%) was the most common in elderly and night (62.6%) in non-elderly patients. Admission rate (odds ratio [OR] 2.512 confidence interval [CI] 2.407–2.621), intensive care unit (ICU) care rate (OR 5.507 [CI] 5.178–5.858), mortality rate (OR 4.593 [CI] 4.086–5.162), and EMR-ISS >25 (OR 5.498 [CI] 5.262–5.745) were compared between patients with alcohol-related injury and non-elderly with non-alcohol-related injury patients. Alcohol consumption in elderly patients results in significant impairment and increases EMR-ISS, ICU care rate, and mortality rate. To reduce injury in elderly patients, alcohol screening, appropriate counseling, and intervention are needed.

## Introduction

Injury is a public health problem accounting for 16% of the global disease burden. Alcohol is an important contributing factor to injury-related emergency department (ED) visits and is the most commonly used and abused substance in the United States. It accounts for one in 10 deaths among adults aged 20–64 years (Fernandez et al., 2019). Several studies have suggested that alcohol consumption is independently associated with injury complications and severity (Draus Jr et al., 2008; Plurad et al., 2011; Rehm et al., 2003).

South Korea has a rapidly aging population. In 2015, 13.2% of South Korea’s population was determined to be ≥65 years old. KOSIS (2016) Park reported that age affects fatality in injury patients (Park et al., 2016). The importance of the association between alcohol use and injury in elderly patients has been recognized (Kowalenko et al., 2013; Selway, Soderstrom & Kufera, 2008).

The role of alcohol in injury patients visiting EDs has not been clearly defined. This study aimed to examine the association of age and alcohol with injury severity and clinical outcomes.

## Materials and Methods

### Study design and setting

This is a retrospective observational study on ED-based injury in-depth surveillance data obtained from the Korea Centers for Disease Control and Prevention, South Korea, which has been prospectively gathering injury-related information nationwide. During the study period, 20 EDs from 2011 to 2014 and 23 EDs from 2015 to 2016 participated in the survey. The hospitals are tertiary academic teaching hospitals across the nation. The data were anonymized before analysis. The Korea Centers for Disease Control and Prevention sends researchers to each hospital who can manage and checkup the data and offer continuous education programs to maintain data quality.

### Study population

This study population included patients who visited EDs between January 2011 and December 2016. Injury patients aged ≥18 years old were included. In South Korea, drinking alcohol is prohibited for those aged <18 years. Therefore, we excluded patients aged <17 years and are not known to be drinking. We defined elderly patients as those aged ≥65 years.

### Data collection and variables

The following variables were collected from electronic medical records based on the definition of variables provided by the Korea Centers for Disease Control and Prevention. We analyzed the following data: patient demographics (age and sex), drinking status (alcohol vs non-alcohol), mode of arrival (walk-in, 119, private ambulance, police, or air transportation), type of insurance (national health insurance, self-pay, vehicle, medicaid beneficiary, private insurance, or work accident), injury-related data (mechanism, place, kind of activity, intentionality, time, and day). Times of ED visit were classified into three categories: day (07:00 to 14:59), evening (15:00 to 22:59), and night (23:00 to 6:59).

Study outcomes were clinical results, rate of admission, intensive care unit (ICU) care, mortality, excess mortality ratio-adjusted injury severity score (EMR-ISS).

### Statistical analysis

Cross-tabulation was performed for non-continuous variables, and Student’s *t*-test was used for continuous variables. A *P*-value of <0.05 was considered statistically significant. To analyze variables associated among alcohol status, age, and outcomes (admission, ICU care, mortality, and EMR-ISS), univariate and multivariate logistic regression analyses were used.

### Ethics statement

This study was approved by the institutional review board (IRB) of Ewha Womans’ University Mok-dong hospital (IRB No. 2019-05-030), and informed consent was waived by the IRB because patient information was anonymized before the analysis.

## Results

**Figure 1:**
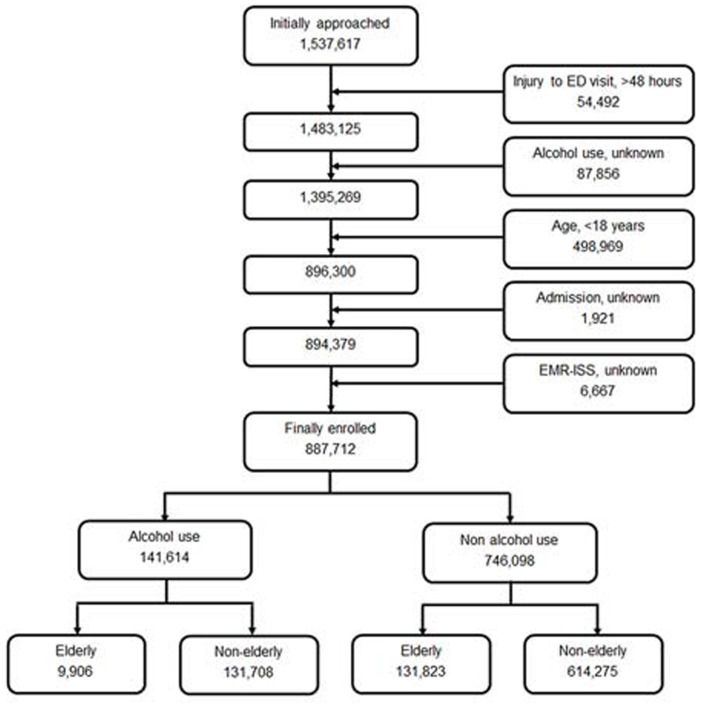
Study flow diagram of enrolled patients.

During the study period, there were 1,537,617 injured patients in the registry, of whom 54,492 patients who visited the ED more than 48 h after injury were excluded; the drinking status of 87,856 patients were not known, and 498,969 were aged <18 years. We also excluded patients who were unknown whether they were admitted to hospital (1,921 patients) and whose EMR-ISS (6,667 patients) were unknown. Finally, we analyzed the data of 887,712 patients. Figure 1 shows the study flow diagram of enrolled patients.

### Demographic data of injury patients at ED in the elderly and non-elderly groups during 2011–2016 (Table 1)

Table 1 shows demographics of injury patients who visited ED during 2011–2016. There were 745,983 (84.0%) non-elderly patients (mean age 40.00 ± 12.95) and 141,729 (16.0%) elderly patients (mean age 74.67 ± 7.04). The number of male patients was 458,594 (61.4%) in non-elderly and 66,481 (46.9%) in elderly patients. The mode of ED visit in non-elderly patients was walk-in in 516,067 (69.2%), 119 in 184,353 (24.7%), and private ambulance in 41,840 (5.6%) patients, where in elderly patients, it was walk-in in 65,389 (46.1%), 119 in 53,782 (37.9%), and private ambulance in 21,845 (15.4%). The proportion of patients who used national health insurance was 75.4%, vehicle insurance was 15.2%, and medicaid beneficiary was 3.3% in injury patients; 141,614 (16.0%) patients who visited the ED due to injury consumed alcohol, and 131,708 (17.7%) of non-elderly and 9,906 (7.0%) of elderly patients had alcohol-related injury.

**Table 1:** Demographic data of injury patients at ED in the elderly and non-elderly groups during 2011–2016.

	Non-elderly (<65 yrs)	Elderly (≥65 yrs)	Total	*p*-value
No. of patients	745,983 (84.0)	141,729 (16.0)	887,712 (100)	
Age (yrs, mean ± SD)	40.0 ± 12.95	74.67 ± 7.04	45.54 ± 17.61	<0.001
Sex				<0.001
Male	458,594 (61.5)	66,481 (46.9)	525,075 (59.1)	
Female	287,389 (38.5)	75,248 (53.1)	362,637 (40.9)	
Mode of arrival				
Walk-in (include car, foot, etc.)	516,067 (69.2)	65,389 (46.1)	581,456 (65.5)	<0.001
119	184,353 (24.7)	53,782 (37.9)	238,135 (26.8)	<0.001
Private ambulance	41,840 (5.6)	21,845 (15.4)	63,694 (7.2)	<0.001
Police	1,720 (0.2)	108 (0.1)	1,828 (0.2)	<0.001
Air	1,204 (0.2)	332 (0.2)	1,536 (0.2)	<0.001
Others	638 (0.1)	230 (0.2)	868 (0.1)	<0.001
Unknown	161 (0.0)	34 (0.0)	195 (0.0)	0.575
Insurance				
National health insurance	559,992 (75.1)	109,145 (77.0)	669,137 (75.4)	<0.001
Self-pay (uninsured)	44,334 (5.9)	4,591 (3.2)	48,925 (5.5)	<0.001
Vehicle	115,967 (15.5)	19,127 (13.5)	135,094 (15.2)	<0.001
Medicaid beneficiary	20,481 (2.7)	8,474 (6.0)	28,955 (3.3)	<0.001
Private insurance	132 (0.0)	25 (0.0)	157 (0.0)	0.989
Work accident	2,595 (0.3)	185 (0.1)	2,780 (0.3)	<0.001
Others	2,268 (0.3)	170 (0.1)	2,438 (0.3)	<0.001
Unknown	214 (0.0)	12 (0.0)	226 (0.0)	<0.001
Alcohol ingestion before injury				<0.001
Yes	131,708 (17.7)	9,906 (7.0)	141,614 (16.0)	
No	614,275 (82.3)	131,823 (93.0)	746,098 (84.0)	

### Injury characteristics of patients at the ED in the elderly and non-elderly groups during 2011–2016 (Table 2)

Table 2 compares the mechanism, place, kind of activity, intentionality, and time of ED presentation between the non-elderly and elderly groups. In injury mechanism, the rate of falls and slips was significantly higher in the elderly (51.4%) than in the non-elderly (22.6%) group. The rate of collision (21.5%) and penetration (14.5%) injuries in non-elderly is higher than that in elderly patients (8.5% and 6.1%, respectively). In elderly patients, the most common place of injury was in the house (44.2%); furthermore, the rate of injury in commercial facilities (13.7% vs 5.1%) and factory, industrial, and construction facilities (8.1% vs 2.0%) was higher in non-elderly than in elderly patients. Elderly patients sustained injuries with daily living activities (49.5%) and leisure activities (17.5), whereas non-elderly patients sustained injuries with daily living activities (32.0%), leisure activities (20.2%), and work (16.3%).

**Table 2:** Injury characteristics of patients at the ED in the elderly and non-elderly groups during 2011–2016.

	Non-elderly (<65 yrs)	Elderly (≥65 yrs)	Total	*p*-value
Mechanism				
Fall, slip	168,255 (22.6)	72,914 (51.4)	241,169 (27.2)	<0.001
Collision	160,157 (21.5)	12,041 (8.5)	172,199 (19.4)	<0.001
Traffic accident	160,190 (21.5)	28,726 (20.3)	188,916 (21.3)	<0.001
Penetration	108,135 (14.5)	8,581 (6.1)	116,716 (13.1)	<0.001
Substance exposure	26,521 (3.6)	6,347 (4.5)	32,868 (3.7)	<0.001
Overuse	29,624 (4.0)	2,916 (2.1)	32,540 (3.7)	<0.001
Drowning, hanging, asphyxia	2,562 (0.3)	814 (0.6)	3,376 (0.4)	<0.001
Thermal injury	17,130 (2.3)	1,218 (0.9)	18,348 (2.1)	<0.001
Machine	9,570 (1.3)	883 (0.6)	10,453 (1.2)	<0.001
Natural disaster	51 (0.0)	15 (0.0)	66 (0.0)	0.134
Others	66,010 (8.0)	6,328 (4.5)	66,338 (7.5)	<0.001
Unknown	3,778 (0.5)	945 (0.7)	4,723 (0.5)	<0.001
Place				
Road	239,594 (32.1)	44,567 (31.4)	284,161 (32.0)	<0.001
Commercial facilities	102,419 (13.7)	7,220 (5.1)	109,639 (12.4)	<0.001
House	209,573 (28.1)	62,614 (44.2)	272,187 (30.7)	<0.001
Outdoor, river, sea	34,180 (4.6)	6,931 (4.9)	41,111 (4.6)	<0.001
Amusement, cultural public facilities	13,473 (1.8)	2,377 (1.7)	15,850 (1.8)	0.001
Transportation area except road	9,504 (1.3)	2,724 (1.9)	12,228 (1.4)	<0.001
Residential facilities	6,696 (0.9)	2,716 (1.9)	9,412 (1.1)	<0.001
Factory, industrial facilities	60,766 (8.1)	2,803 (2.0)	63,569 (7.2)	<0.001
Farm	8,388 (1.1)	3,442 (2.4)	11,830 (1.3)	<0.001
School, education facilities	6,633 (0.9)	160 (0.1)	6,793 (0.8)	<0.001
Sport facilities	32,876 (4.4)	981 (0.7)	33,857 (3.8)	<0.001
Medical facilities	12,059 (1.6)	3,879 (2.7)	15,938 (1.8)	<0.001
Others	641 (0.1)	134 (0.1)	775 (0.1)	<0.001
Unknown	9,181 (1.2)	1,811 (0.8)	10,362 (1.2)	<0.001
Activity				
Leisure activities	150,961 (20.2)	24,865 (17.5)	175,726 (19.8)	<0.001
Daily living activities	238,731 (32.0)	70,129 (49.5)	308,860 (34.8)	<0.001
Unpaid labor	103,240 (13.8)	23,729 (16.7)	126,969 (14.3)	<0.001
Work	121,689 (16.3)	10,258 (7.2)	131,947 (14.9)	<0.001
Exercise	35,111 (4.7)	1,785 (1.3)	36,896 (4.2)	<0.001
Travel	3,419 (0.5)	453 (0.3)	3,872 (0.4)	<0.001
Hospital treatment	2,036 (0.3)	1,863 (1.3)	3,899 (0.4)	<0.001
Education	2,739 (0.4)	27 (0.0)	2,766 (0.3)	<0.001
Others	83,212 (11.2)	7,675 (5.4)	90,884 (10.2)	<0.001
Unknown	4,845 (0.6)	1,045 (0.7)	5,890 (0.7)	<0.001
Intentionality				
Unintentional	664,108 (89.0)	134,390 (94.8)	798,498 (90.0)	<0.001
Assault	53,541 (7.2)	2,078 (1.5)	55,619 (6.3)	<0.001
Self-harm, suicide	25,167 (3.4)	4,500 (3.2)	29,667 (3.3)	<0.001
Others	906 (0.1)	204 (0.1)	1,110 (0.1)	0.028
Unknown	2,261 (0.3)	557 (0.4)	2,818 (0.3)	<0.001
Day of presentation				<0.001
Weekday (Mon-Thu)	355,599 (47.7)	75,902 (53.6)	431,501 (48.6)	
Weekend (Fri-Sun)	390,384 (52.3)	65,827 (46.4)	456,211 (51.4)	
Time of presentation				
Day (7∼14 h)	204,742 (27.4)	59,076 (41.7)	263,818 (29.7)	<0.001
Evening (15∼22 h)	335,621 (45.0)	66,125 (46.7)	401,746 (45.3)	<0.001
Night (23∼6 h)	205,606(27.6)	16,526 (11.7)	222,132 (25.0)	<0.001

### General characteristics of injury patients in the elderly and non-elderly groups who consumed alcohol (Table 3)

We analyzed injury patients who consumed alcohol. There were 74.6% males (73.8% non-elderly/85.70% elderly). These patients had a higher rate of visiting EDs by 119 ambulance (43.9% non-elderly/61.0% elderly). Around 5.0% availed medicaid beneficiary (4.8% non-elderly/8.0% elderly).

### Injury characteristics in the elderly and non-elderly groups who consumed alcohol (Table 4, Figs. 2 and 3)

Table 4 compares the mechanism, place, kind of activity, intentionality, and time of ED presentation between non-elderly and elderly group who consumed alcohol. Fall and slips are the most common injury mechanism (37.9%) in these patients (36.3% non-elderly/58.40% elderly).

**Table 3:** General characteristics of injury patients in the elderly and non-elderly groups who consumed alcohol.

	Non-elderly (<65 yrs)	Elderly (≥65 yrs)	Total	*p*-value
No. of patients	131,708 (93.0)	9,906 (7.0)	141,614 (100)	
Age	38.98 ± 12.62	71.50 ± 5.36	41.25 ± 14.80	<0.001
Sex				<0.001
Male	97,176 (73.8)	8,493 (85.7)	105,669 (74.6)	
Female	34,532 (26.2)	1,413 (14.3)	35,945 (25.4)	
Mode of arrival				
Walk-in (include car, foot, etc.)	65,227 (49.5)	2,524 (25.5)	67,751 (47.8)	<0.001
119	57,773 (43.9)	6,047 (61.0)	63,820 (45.1)	<0.001
Private ambulance	7,570 (5.7)	1,280 (12.9)	8,850 (6.2)	<0.001
Police	925 (0.7)	28 (0.3)	953 (0.7)	<0.001
Air	93 (0.1)	19 (0.2)	112 (0.1)	<0.001
Others	88 (0.1)	5 (0.1)	93 (0.1)	0.540
Unknown	32 (0.0)	3 (0.0)	35 (0.0)	0.715
Insurance				
National health insurance	103,975 (78.9)	8,064 (81.4)	112,039 (79.1)	<0.001
Self-pay (uninsured)	12,702 (9.6)	596 (6.0)	13,298 (9.4)	<0.001
Vehicle	8,193 (6.2)	434 (4.4)	8,627 (6.1)	<0.001
Medicaid beneficiary	6,299 (4.8)	789 (8.0)	7,088 (5.0)	<0.001
Private insurance	24 (0.0)	3 (0.0)	27 (0.0)	0.402
Work accident	23 (0.0)	1 (0.0)	24 (0.0)	0.587
Others	416 (0.3)	16 (0.2)	432 (0.3)	0.007
Unknown	76 (0.1)	3 (0.0)	79 (0.1)	0.265

**Table 4:** Injury characteristics in the elderly and non-elderly groups who consumed alcohol.

	Non-elderly (<65 yrs)	Elderly (≥65 yrs)	Total	*p*-value
Mechanism				
Fall, slip	47,861 (36.3)	5,789 (58.4)	53,650 (37.9)	<0.001
Collision	41,992 (31.9)	1,048 (10.6)	43,040 (30.4)	<0.001
Traffic accident	13,658 (10.4)	1,073 (10.8)	14,731 (10.4)	0.146
Penetration	12,576 (9.5)	238 (2.4)	12,814 (9.0)	<0.001
Substance exposure	9,077 (6.9)	1,292 (13.0)	10,369 (7.3)	<0.001
Overuse	1,236 (0.9)	33 (0.3)	1,269 (0.9)	<0.001
Drowning, hanging, asphyxia	836 (0.6)	62 (0.6)	898 (0.6)	0.915
Thermal injury	751 (0.6)	18 (0.2)	769 (0.5)	<0.001
Machine	60 (0.0)	8 (0.1)	68 (0.0)	0.123
Natural disaster	5 (0.0)	0 (0.0)	5 (0.0)	0.540
Others	1,499 (1.1)	71 (0.7)	1,570 (1.1)	<0.001
Unknown	2,157 (1.6)	274 (2.8)	2,431 (1.7)	<0.001
Place				
Road	48,487 (36.8)	4,026 (40.6)	52,513 (37.1)	<0.001
Commercial facilities	41,254 (31.3)	1,176 (11.9)	42,430 (30.0)	<0.001
House	28,506 (21.6)	3,349 (33.8)	31,855 (22.5)	<0.001
Outdoor, river, sea	3,434 (2.6)	342 (3.5)	3,776 (2.7)	<0.001
Amusement, cultural public facilities	2,533 (1.9)	200 (2.0)	2,733 (1.9)	0.504
Transportation area except road	1,747 (1.3)	378 (3.8)	2,125 (1.5)	<0.001
Residential facilities	1,127 (0.9)	119 (1.2)	1,246 (0.9)	<0.001
Factory, industrial facilities	591 (0.4)	28 (0.3)	619 (0.4)	0.016
Farm	298 (0.2)	50 (0.5)	348 (0.2)	<0.001
School, education facilities	322 (0.2)	3 (0.0)	325 (0.2)	<0.001
Sport facilities	302 (0.2)	12 (0.1)	314 (0.2)	0.027
Medical facilities	157 (0.1)	36 (0.4)	193 (0.1)	<0.001
Others	99 (0.1)	9 (0.1)	108 (0.1)	0.585
Unknown	2,851 (2.2)	178 (1.8)	3,029 (2.1)	0.015
Activity				
Leisure activities	41,853 (31.8)	3,486 (35.2)	45,339 (32.0)	<0.001
Daily living activities	25,840 (19.6)	2,789 (28.2)	28,629 (20.2)	<0.001
Unpaid labor	13,987 (10.6)	1,235 (12.5)	15,222 (10.7)	0.129
Work	1,345 (1.0)	117 (1.2)	1,462 (1.0)	<0.001
Exercise	286 (0.2)	10 (0.1)	296 (0.2)	0.015
Travel	187 (0.1)	8 (0.1)	195 (0.1)	0.113
Hospital treatment	53 (0.0)	15 (0.2)	68 (0.0)	<0.001
Education	48 (0.0)	3 (0.0)	51 (0.0)	0.755
Others	46,538 (35.3)	2,053 (20.7)	48,591 (34.3)	<0.001
Unknown	1,571 (1.2)	190 (1.9)	1761 (1.2)	<0.001
Intentionality				
Unintentional	86,063 (65.3)	7,913 (79.9)	93,976 (66.4)	<0.001
Assault	31,201 (23.7)	618 (6.2)	31,819 (22.5)	<0.001
Self-harm, suicide	12,825 (9.7)	1,192 (12.0)	14,017 (9.9)	<0.001
Others	328 (0.2)	37 (0.4)	365 (0.3)	0.018
Unknown	1,291 (1.0)	146 (1.5)	1,437 (1.0)	<0.001
Day of presentation				<0.001
Weekday (Mon-Thu)	63,372 (48.1)	5,233 (52.8)	68,605 (48.4)	
Weekend (Fri-Sun)	68,336 (51.9)	4,673 (47.2)	73,009 (51.6)	
Time of presentation				
Day (7∼14 h)	17,291 (13.1)	1,555 (15.7)	18,846 (13.3)	<0.001
Evening (15∼22 h)	31,941 (24.3)	6,019 (60.8)	37,960 (26.8)	<0.001
Night (23∼6 h)	82,468 (62.6)	2,332 (23.5)	141,606 (59.9)	<0.001

Place of injury was the roads (40.6%), around the house (33.8%), and commercial facilities (11.9%) in elderly patients and roads (36.8%), commercial facility (31.3%), and around the house (21.6%) in non-elderly patients. Activities that led to injury was leisure activities (35.2%) and daily living activities (28.2%) in elderly patients and leisure activities (31.8%) and daily living activities (19.6%) in non-elderly patients. On the intentionality of injury, self-harm and suicide rates were 12.0% in elderly and 9.7% in non-elderly patients.

**Figure 2:**
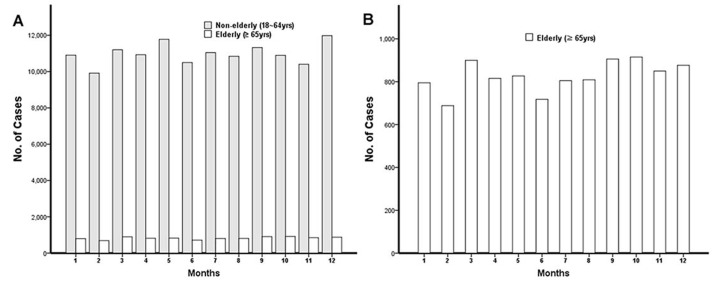
Number of patients who visit ED by month. (A) The monthly injury incidence of non-elderly and elderly patients. (B) The monthly injury incidence of elderly patients.

**Figure 3:**
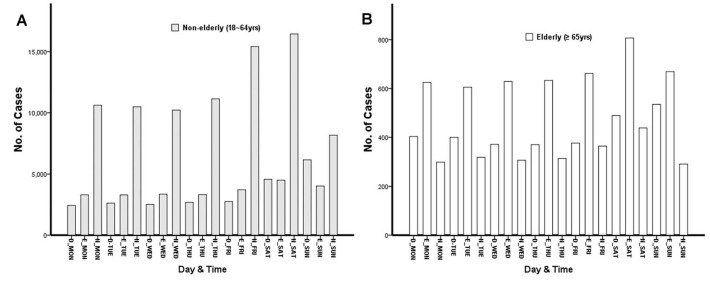
Number of patients who visit ED by day and time. (A) The time of injury incidence in non-elderly patients. (B) The time of injury incidence in elderly patients.

Injury was more common in weekdays (52.8%) in elderly patients and in weekends (52.9%) in non-elderly patients. The monthly incidence of injury showed no significant difference between elderly and non-elderly patients (Fig. 2). With respect to the time of injury, evening (60.8%) was the most common in elderly and night (62.6%) in non-elderly patients (Fig. 3).

### Comparison of clinical outcomes and injury severity between non-elderly and elderly patients by alcohol use (Table 5)

We divided the study group by age and alcohol use. In the alcohol use group, the elderly had a higher ICU care rate (odds ratio [OR] 3.065 confidence interval [CI] 2.872–3.727), mortality rate (OR 5.136 [CI] 4.501–5.861), and EMR-ISS (≥25) (OR 2.535 [CI] 2.249–2.462). In the non-alcohol use group, the elderly had a higher ICU care rate (OR 2.709 [CI] 2.637–2.783), mortality rate (OR 4.456 [CI] 4.266–4.653), and EMR-ISS (≥25) (OR 2.541 [CI] 2.497–2.538).

**Table 5:** Comparison of clinical outcomes and injury severity between non-elderly and elderly patients by alcohol use.

	Non-elderly (<65 yrs)	Elderly (≥65 yrs)	*p*-value	OR (95% CI)
**Alcohol use**				
Admission	22,091 (16.8)	3,233 (32.6)	<0.001	2.404 (2.300–2.513)
ICU	5,853 (4.4)	1,236 (12.5)	<0.001	3.065 (2.872–3.272)
Mortality	826 (0.6)	311 (3.1)	<0.001	5.136 (4.501–5.861)
EMR-ISS (≥25)	20,568 (15.6)	3,005 (30.3)	<0.001	2.353 (2.249–2.462)
**No alcohol use**				
Admission	99,317 (16.2)	53,117 (40.3)	<0.001	3.499 (3.454–3.545)
ICU	15,500 (2.5)	8,638 (6.6)	<0.001	2.709 (2.637–2.783)
Mortality	4,305 (0.7)	4,019 (3.0)	<0.001	4.456 (4.266–4.653)
EMR-ISS (≥25)	45,080 (7.3)	22,081 (16.8)	<0.001	2.541 (2.497–2.585)

### Logistic regression for interaction between clinical outcomes and age and alcohol use in injured patients (Table 6)

Statistically significant interaction effect was seen between age and alcohol use. To evaluate the effect of age and alcohol, we divided the study population into non-elderly, non-alcohol use group (G1), non-elderly, alcohol use group (G2), elderly, non-alcohol use group (G3), and elderly, alcohol use group (G4). Logistic regression revealed statistically significant differences among the four groups in admission rate, ICU care rate, mortality rate, and EMR-ISS. Table 6 shows ORs and 95% CI with G1 as the reference. Compared with G1, the OR and CI in G2, G3, and G4 were OR 1.045 [CI] 1.028–1.062, OR 3.499 [CI] 3.454–3.545, and OR 2.512 [CI] 2.407–2.621, respectively, for admission rate; OR 1.797 [CI] 1.742–1.853, OR 2.709 [CI] 2.637–2.783, and OR 5.507 [CI] 5.178–5.858, respectively, for ICU care rate; OR 0.894 [CI] 0.830–0.964, OR 4.456 [CI] 4.266–4.653, and OR 4.593 [CI] 4.086–5.162, respectively, for mortality rate; OR 2.337 [CI] 2.296–2.378, OR 2.541 [CI] 2.497–2.585, and OR 5.498 [CI] 5.262–5.745, respectively, for EMR-ISS >25(Kim et al., 2009). G4 had higher OR for EMR-ISS, ICU care rate, and mortality rate.

**Table 6:** Logistic regression for interaction between clinical outcomes and age and alcohol use in injured patients.

	OR	95% CI	*p*-value
**Admission**			
Non-elderly, no alcohol use	1.000	Reference	–
Non-elderly, alcohol use	1.045	1.028–1.062	<0.001
Elderly, no alcohol use	3.499	3.454–3.545	<0.001
Elderly, alcohol use	2.512	2.407–2.621	<0.001
**ICU**			
Non-elderly, no alcohol use	1.000	Reference	–
Non-elderly, alcohol use	1.797	1.742–1.853	<0.001
Elderly, no alcohol use	2.709	2.637–2.783	<0.001
Elderly, alcohol use	5.507	5.178–5.858	<0.001
**Mortality**			
Non-elderly, no alcohol use	1.000	Reference	–
Non-elderly, alcohol use	0.894	0.830–0.964	0.003
Elderly, no alcohol use	4.456	4.266–4.653	<0.001
Elderly, alcohol use	4.593	4.086–5.162	<0.001
**EMR-ISS** (≥**25**)			
Non-elderly, no alcohol use	1.000	Reference	–
Non-elderly, alcohol use	2.337	2.296–2.378	<0.001
Elderly, no alcohol use	2.541	2.497–2.585	<0.001
Elderly, alcohol use	5.498	5.262–5.745	<0.001

## Discussion

This study shows that alcohol-related injury had serious clinical outcomes in elderly patients. The relationship between alcohol consumption and injury has already been revealed by various studies and analyses. Acute alcohol consumption, even in small amounts, increases the risk of injury (Cherpitel, 2007). Globally, 10%–18% of injuries that result in ED visits have been reported to be related to alcohol consumption (WHO, 2007). In the elderly population, injury has important public health implications. Injured elderly patients have a higher mortality rate than younger adults (Champion et al., 1989), and it has been reported that relatively mild mechanisms may cause injury in the elderly (Giannoudis et al., 2009). This study aimed to investigate the relationship between alcohol consumption and injury in elderly patients and to analyze the effect of alcohol on injury severity.

The rate of alcohol consumption among elderly patients with injury was 7.0% (Table 1). Ekeh et al. (2014) reported that 11.1% of injury patients aged ≥65 years who visited the level 1 trauma center showed positive blood alcohol content (BAC). In another study, the BAC of 12.6% of injury patients aged ≥65 years who visited the ED was >100 mg/dl. (Rivara et al., 1993) The difference between previous studies and the present study is probably due to the different alcohol-drinking behaviors of each region and cultures.

The number of males in the alcohol injury elderly group was higher than that in the younger adult group (Table 3). This is thought to be due to differences in alcohol-drinking behavior based on age and sex. According to Kim’s analysis of data from the Korea National Health and Nutrition Examination Survey, prevalence of intermediate risk drinking (alcohol use disorder identification test score of ≥8) was 23.1%, 13.5%, and 4.2% for females aged 19–44 years, 45–64 years, and ≥65 years, respectively, and 59.7%, 60.1%, and 36.7%, respectively, for males (Hong, Noh & Kim, 2017). In elderly females, the drinking rate was significantly lower; this explains why the proportion of males in the elderly alcohol injury patients is significantly higher. It can also be seen that the rate of alcohol consumption is closely related to the occurrence of alcohol-related injury.

The rate of using 119 in the alcohol injury elderly group was higher than that in the total elderly injury group (Tables 1 and 3). A total of 119 of Korea, regardless of the severity, sends the patient to a hospital free of charge, if they request the emergency medical system. For this reason, 119 is more likely to be used when patients cannot move by themselves or in poor economic conditions. This tendency is prominent in the elderly. (Kang, 2015) Besides, the higher use of 119 in the drinking elderly group than in the total elderly injury group can be attributed to the increased severity of injury due to alcohol (Tables 4 and 5). At a time when the society is rapidly aging, this will lead to an increase in medical costs.

The percentage of falls and slips in the elderly alcohol group was higher than that in the total elderly injury group. Falls and slips are the most common and one of the major mechanisms in elderly injury. They can result in fractures, immobility, and reduced daily living activities, even leading to death (WHO, 2017). Risk factors for elderly falls include age, drug use, cognitive impairment, and sensory deficit (Fuller, 2000). Even though there are various opinions as to whether alcohol is a risk factor in elderly falls (Adams & Jones, 1998), studies have suggested that alcohol is a risk factor for the occurrence of and mortality due to elderly falls (Schick et al., 2018; Sorock et al., 2006). In this study, the incidence of falls and slips was higher in the elderly alcohol group, suggesting that alcohol contributes to the occurrence of falls.

In this research, intentional injury in patients who consumed alcohol was higher than in total injury patients. A total of 9.9% of patients who consumed alcohol had intentions of self-harm or suicide, whereas 12.0% of elderly patients who consumed alcohol attempted suicide and self-harm. Darvishi et al. (2015) reported that alcohol use significantly increases the risk of suicidal ideation, suicide attempt, and completed suicide. Compared with the total elderly injury group, intentional injury (assault, self-harm/suicide) accounted for a large proportion of the alcohol elderly injury group (Tables 2 and 4). Therefore, alcohol can be seen as one of the factors causing intentional injury. In the alcohol injury group, the ratio of unintentional injury and self-harm/suicide was higher in the elderly group than in the younger group. This is because of the higher rate of violence in the younger alcohol group. According to Dinh et al. (2014), the proportion of alcohol-related assault patients who visited the trauma center was 3%, 52%, and 21% in those aged ≥65 years, 24–44 years, and 45–64 years old, respectively.

ED visits in elderly injury patients were the most frequent during the evening, followed by day, then night (Table 2). On the other hand, the order was evening, night, and day in elderly alcohol injury patients (Table 4). This is related to the time of day when alcohol drinking occurs. In the younger alcohol injury group, visits were the highest during the night, (Table 4) suggesting a difference in the time zone in which alcohol consumption is done in different age groups.

With regard to injury severity, age and drinking were found to have interaction effects. Injury severity was positively correlated with age and alcohol consumption. Particularly, this was observed in ICU care rate and EMR-ISS score of ≥25 in severely injured patients (Tables 5 and 6). Although there is no doubt that alcohol is a risk factor in causing damage, various studies have shown whether alcohol consumption is related to the severity of the damage. Some studies have reported that alcohol consumption and injury severity are not significantly correlated (Ahmed & Greenberg, 2019; Schneiders et al., 2017). There was a difference according to the mechanism of damage. (Valdez et al., 2016) It has also been reported that the effect of alcohol is different depending on whether the BAC is 400 or more (Afshar et al., 2016). While positive BACs have been shown to reduce mortality rate in trauma patients (Yaghoubian et al., 2009), drinking has been reported to be related to injury severity (Elshiere, Noorbhai & Madiba, 2017; Swearingen et al., 2010). This study showed that age and drinking had an interaction effect and were associated with the occurrence of severe injury.

There are some limitations. This study used data obtained from the Emergency Department-Based Injury In-depth Surveillance conducted by the Korea Centers for Disease Control and Prevention at 23 EDs to prevent injury. However, one of the limitations of this study is that information on alcohol consumption behavior required to understand the relationship between injury and drinking is limited, and the exact time interval between alcohol consumption and injury is unknown. Additionally, the fact that alcohol consumption was based on the patient’s self-report was a limitation to objectively determine whether the patient was drinking or not. In England, alcohol screening with referral or intervention is becoming part of routine practice. And the rate of alcohol identification and intervention in ED has had improvements (Patton & Green, 2018). However, there is no system for alcohol screening and intervention in Korea. 23 EDs supported by KCDC gather the information whether the injury patients drink or not. Korean EDs need to screen alcohol with appropriate tools such as AUDIT score for future study and public health.

Tests to reveal blood alcohol level were not performed. Zautcke et al. (2002) also reported that falls are the most common mechanism of injury in elderly patients who under the influence of alcohol. They performed blood alcohol level test in 5.2% of geriatric patients and obtained positive results for 49.7% (Zautcke et al., 2002). Csipke et al. (2007) reported a low correlation with BAC and alcohol questionnaire results. This suggests that not only alcohol screening but also an objective BAC measurement should be performed. Alcohol screening and BAC information should be included in the future study. And More prospective studies are needed to investigate true alcohol level. Also, there is a possibility of selection biases due to unknown data on alcohol use, admission rate, and EMR-ISS.

## Conclusions

Elderly alcohol injury patients increased the incidence of falls, the most common injury mechanism in elderly, and increased the utilization rate of 119. Moreover, age and alcohol were risk factors for ICU care and EMR-ISS score of ≥25. Alcohol screening and intervention is required in geriatric trauma patients because alcohol use is significantly prevalent in trauma (Ekeh et al., 2014). This study shows that alcohol-related injury had serious clinical outcomes. Therefore, alcohol screening and intervention in ED are needed to prevent alcohol-related injury in the elderly.

## Additional Information and Declarations

### Data Availability

The following information was supplied regarding data availability: The Korean Center for Disease Control (KCDC) is the authority for accessing the data analyzed, and there are ethical restrictions on sharing a dataset because the data contain potentially identifying information. The KCDC (http://www.cdc.go.kr) can be contacted for data access via the Injury research team email (kcdcinjury@korea.kr) or by calling 82-43-719-7407.
